# Effect of Calcium Hydroxide on Physicochemical and In Vitro Digestibility Properties of Tartary Buckwheat Starch-Rutin Complex Prepared by Pre-Gelatinization and Co-Gelatinization Methods

**DOI:** 10.3390/foods12050951

**Published:** 2023-02-23

**Authors:** Xinxin Ding, Xiaoping Li, Qiling Cai, Zhen Ma, Tian Ren, Xinzhong Hu

**Affiliations:** College of Food Engineering and Nutritional Science, Shaanxi Normal University, Xi’an 710119, China

**Keywords:** Tartary buckwheat, starch-rutin complex, calcium hydroxide, pre-gelatinization, co-gelatinization, physicochemical properties, digestibility

## Abstract

This study examined the effect of calcium hydroxide (Ca(OH)_2_, 0.6%, *w*/*w*) on structural, physicochemical and in vitro digestibility properties of the complexed system of Tartary buckwheat starch (TBS) and rutin (10%, *w*/*w*). The pre-gelatinization and co-gelatinization methods were also compared. SEM results showed that the presence of Ca(OH)_2_ promoted the connection and further strengthened the pore wall of the three-dimensional network structure of the gelatinized and retrograded TBS-rutin complex, indicating the complex possessed a more stable structure with the presence of Ca(OH)_2_, which were also confirmed by the results of textural analysis and TGA. Additionally, Ca(OH)_2_ reduced relative crystallinity (RC), degree of order (DO) and enthalpy, inhibiting their increase during storage, thereby retarding the regeneration of the TBS-rutin complex. A higher storage modulus (G′) value was observed in the complexes when Ca(OH)_2_ was added. Results of in vitro digestion revealed that Ca(OH)_2_ retarded the hydrolysis of the complex, resulting in an increase in values in slow-digestible starch and resistant starch (RS). Compared with pre-gelatinization, the complex process prepared with the co-gelatinization method presented lower RC, DO, enthalpy, and higher RS. The present work indicates the potential beneficial effect of Ca(OH)_2_ during the preparation of starch-polyphenol complex and would be helpful to reveal the mechanism of Ca(OH)_2_ on improving the quality of rutin riched Tartary buckwheat products.

## 1. Introduction

Tartary buckwheat is a kind of crop of the family Polygonaceae and genus *Fagopyrum,* which is broadly cultivated and occupies a crucial part in Europe and Asia [1,2]. Compared with common buckwheat, Tartary buckwheat has a higher concentration of polyphenols, so it has higher nutritional value and health function. As a kind of medicine and food homologous material, Tartary buckwheat has been confirmed as a good source of rutin [2]. Rutin is reported to have antioxidant, anti-inflammatory, anti-hypertensive, and anti-obesity activity properties and reduces the risk of arteriosclerosis [1,3], which endow Tartary buckwheat with multiple health benefits, such as the prevention and treatment of diabetes, hypertension, and a variety of other chronic diseases, which has attracted the increasing interest of consumers and food scientists [4]. In addition, Tartary buckwheat is rich in starch, and its content has been reported to be more than 70% of the total dry weight, which will affect the edible quality of whole buckwheat products [5]. In recent years, there has been renewed attention to Tartary buckwheat when researchers found that polyphenols can interact with starch to improve its stability and bioavailability and simultaneously change the structure and digestibility of starch in response to different processing methods.

The interaction between polyphenols and starch has been widely reported. The study found that quercetin had an effect on the morphological structure and thermal characteristics of pre-gelatinized starch; the addition of quercetin greatly reduced digestibility [6]. He et al. [7] chose rutin to co-cook with TBS and discovered that appropriate concentrations of rutin could affect the digestive and physicochemical characteristics of TBS, and they can also inhibit the retrogradation of TBS. In recent years, it has been discovered that the starch-polyphenol complex can be formed by hydrogen bonds, hydrophobic interaction, van der Waals force, and electrostatic interaction [8]. The complexation of starch with polyphenols can decrease the digestibility, increase the resistant starch fractions, improve the stability of polyphenols, and regulate the release of polyphenols [8]. However, these beneficial impacts of complexation on starch and polyphenols are related to processing methods [8]. Pre-gelatinization was considered the most popular physical preparation method of starch- polyphenols complex [9], which might be that pre-gelatinization of starch caused starch granules to be “destructured” and converted to random coils before polyphenols were added [8]. Although the pre-gelatinization process is similar to cooking, hence safe and time-saving [9], in the actual processing of raw materials rich in starch and polyphenols, starch and polyphenols are often co-gelatinized. However, little literature has clarified the differences between starch-polyphenols complex prepared with pregelatinized and co-gelatinized based on their physicochemical and in vitro digestibility properties. These differences may help in understanding the change mechanism of nutrition and eating qualities during actual product processing.

Tartary buckwheat is mostly consumed as noodles, steam buns, pancakes, and muffins in the main cultivation countries, such as China, Ukraine, Japan, etc. [1]. However, Tartary buckwheat has a low gluten concentration, which results in poor processing properties for pure Tartary buckwheat powder. Previously, many processing methods, including microwave, gaseous ozone, and heat-moisture treatment, have been made to modify the properties of Tartary buckwheat starch (TBS) so as to improve the quality of noodles [10]. “Hele,” a popular traditional Chinese food, is made from pure Tartary buckwheat flour by adding Ca(OH)_2_. During the manufacturing process, adding Ca(OH)_2_ will raise the calcium content of noodles and promote the formation of the calcium-modified starch complex, which could be useful for enhancing the physicochemical properties of products [11]. In many food processing processes, Ca(OH)_2_ is also added to improve food quality characteristics. Palacios [12] discovered that calcium affects the physicochemical characteristics of starch granules and that the granule surface changes or even becomes porous after treatment. And the Nixonian hot alkaline method is utilized throughout Mexico and Central America to produce tortillas and instant corn meal, indicating that the presence of calcium ions is beneficial to improve the flavor and nutritional characteristics of foods [13]. Our previous research also demonstrated that the quality characteristics of buckwheat noodles could be improved by adding Ca(OH)_2_, while these changes have not been explained from the perspective of the interaction between starch and calcium [10].

Different processing methods may lead to differences in the composition and content of phenolic compounds in Tartary buckwheat products, but there is no comparative study between pre-gelatinization and co-gelatinization. Moreover, the mechanism of action between Ca(OH)_2_-starch and rutin is still unclear. Therefore, to evaluate how Ca(OH)_2_ and rutin affect the physicochemical characteristics of starch during processing, Ca(OH)_2_ and rutin interacted with TBS under different heating modes (pre-gelatinization and co-gelatinization). This research is expected to provide a new method for preparing starch-polyphenol complexes and be helpful in revealing the possible role and mechanism of Ca(OH)_2_ during the processing of Tartary buckwheat.

## 2. Materials and Methods

### 2.1. Materials

Tartary buckwheat flour was purchased from Saixue Grain & Oil Industry and Trade Co., Ltd. (Dingbian, Shaanxi, China). Rutin was provided by Shanghai Yuanye Bio-Technology Co., Ltd. (Shanghai, China). Calcium hydroxide (food grade) was purchased from Guangzhou JIAYE food ingredients Co., Ltd. (Guangzhou, China). Porcine pancreatin was purchased from Solarbio Science &Technology Co., Ltd. (4000 U/mL, P7304, Beijing, China). Amyloglucosidase from *Aspergillus niger* was supplied by Aladdin Industrial Corporation (1 × 104 U/mL, E1619059, Shanghai, China).

### 2.2. Extraction of Starch

According to a prior study [10], the TBS was isolated from the Tartary buckwheat flour. Tartary buckwheat flour (600 g) was soaked into 0.15% aqueous sodium hydroxide solution (3 L) for 16 h at 25 °C, agitated with a magnetic agitator, and centrifuged at 4000 rpm for 10 min after being sieved through a 100-mesh screen. We reserved the white precipitate and washed the precipitate with 80% ethanol and deionized water until the precipitated surface became white. It was then dried at 40 °C for 48 h and, after 150-mesh screening, stored at 4 °C.

### 2.3. Preparation of Pre-Gelatinized Complexes

Pre-gelatinized complexes were prepared through previous literature [6] with some modifications. 3 g of TBS (dry basis) dissolved in 40 mL distilled water or Ca(OH)_2_ solution (0.6%, based on TBS weight), the mixtures were stirred at room temperature for 10 min before being heated at 90 °C for 10 min with constant stirring at 150 rpm. Subsequently, 10 mL of rutin solution (0, 10.0%, based on TBS weight) was added into the pre-gelatinized starch solution, and we continued heating for 20 min with stirring at 150 rpm. Paste liquids were placed at room temperature for 1 h to obtain pre-gelatinized samples, marked for pre-TBSR and pre-TBSRCa; in order to get the retrograded sample, the pastes were kept at 4 °C (1 day) and labeled as pre-TBSR-1d and pre-TBSRCa-1d. After freeze-drying, all samples were ground and passed through 100 mesh sieves for further assessment.

### 2.4. Preparation of Co-Gelatinized Complexes

Co-gelatinized complexes of TBS, Ca(OH)_2_ and rutin complexes were prepared according to the previous literature [2] with some modifications. Three grams of TBS (dry basis) were added with rutin (0, 10.0%, based on TBS weight) dispersed in Ca(OH)_2_ solution (0, 0.6%, based on TBS weight); the mixtures were stirred for 10 min at room temperature before being heated at 90 °C for 30 min with constant stirring at 150 rpm. Paste liquids were placed at room temperature for 1 h to obtain the co-gelatinization samples, marked for GTBS, GTBSCa, TBSR, and TBSRCa, in order to get the retrograded sample, the pastes were kept at 4 °C (1 day) and labeled as GTBS-1d, GTBSCa-1d, TBSR-1d, and TBSRCa-1d. After freeze-drying, all samples were ground and passed through 100 mesh sieves for further assessment.

### 2.5. Solubility and Swelling Power (SP)

The solubility and SP of samples were evaluated using a previous method [14]. Fifty mg powder (W_1_) was put into pre-weighed centrifuge tubes, added 5 mL distilled water and stirred continuously for 30 min at 90 °C. After cooling to room temperature, centrifuged at 4000 rpm for 20 min. Separate the sediment (W_2_) and supernatant, transfer the supernatant to a pre-weighed glass beaker and dried at 105 °C to constant weight (W_3_). The following formulas were used to determine the solubility and SP:

$$\mathrm{Solubility}\left(g/100g\right)=\frac{W3}{W1}\times 100$$

$$\mathrm{SP}\left(g/100g\right)=\frac{W2}{W1-W3}\times 100$$

### 2.6. Scanning Electron Microscopy (SEM)

Morphological characteristics of starch and complexes were analyzed through SEM (Quanta 200, FEI Company, Hillsboro, OR, USA) at 15.0 kV. The sample was stabilized on the surface of copper stubs before being thinly coated with gold, then observed under 20 kV.

### 2.7. Gel Firmness (TPA)

The starch (or complex) pastes were prepared according to the instructions in Section 2.3 and Section 2.4. Heated paste liquids were loaded into 50 mL centrifuge tubes and kept at 4°C for 24 h to obtain retrograded samples, which were then sliced into cylinders with a thickness of 1 cm each. A texture analyzer (CT3, Brookfield, Middleborough, MA, USA) was used to measure the gel firmness with a P/36 probe at 1.0 mm/s.

### 2.8. Thermogravimetry Analysis (TGA)

The TGA of all samples was measured using a Thermoanalyzer System (Q1000DSC + LNCS + FACS Q600SDT, TA Instruments, New Castle, DE, USA) following the procedure proposed by Gao et al. [15]. Briefly, the samples (5.0 mg, dry basis) were weighted into an aluminum crucible, and measurements were made in the temperature range of 25–600 °C at a heating rate of 10 °C/min under a dynamic atmosphere of dry nitrogen flowing at a rate of 20 mL/min.

### 2.9. X-ray Diffraction (XRD)

An X-ray diffractometer (D8 Advance, Bruker Inc., Falkenried, Germany) was used to determine the crystal structures of all samples. The diffractometer worked at 40 kV and 100 mA with scanning radiation in the angular range of 4°–40° (2θ) at a scanning speed of 0.1°/s. Jade software version 6.0 was used to analyze the relative crystallinity of each powder.

### 2.10. Fourier Transform Infrared Spectroscopy (FT-IR)

The sample and KBr powder were mixed (1:100, *w*/*w*) and equilibrated in an oven at 40 ° C; after grinding, we pressed the mixed power into sheets with a vacuum compressor. The FT-IR spectrometer (Bruker GmBH, Ettlingen, Germany) was used to determine the spectral data of all samples through 32 scans in a band with a texture number range of 400–4000 cm^−1^.

### 2.11. Differential Scanning Calorimetry (DSC)

Accurately weighed 3 mg (dry basis) of sample and added into 9 uL of distilled water, placed in aluminum pans before analysis. All samples were measured by differential scanning calorimeter (DSC1, Mettler Toledo Corp, Columbus, OH, USA) as presented by Yu [16]. The DSC pan was heated from 20 °C to 120 °C at a rate of 10 °C/min while being shielded by nitrogen at a flow rate of 20 mL/min.

### 2.12. Rheological Properties

A rheometer (MCR 302, Anton Par, Austria) equipped with a parallel plate geometry and a probe (diameter = 25 mm, gap = 1 mm) was used to conduct steady shear and frequency sweep investigations on all samples. All sample suspensions (1.5 mL) were deposited onto the metal platform in accordance with the previously reported procedure [15]. All the samples were analyzed at 25 °C with shear rates ranging from 0.1 to 300 s^−1^ and subsequently from 300 to 0.1 s^−1^ at a constant shear stress of 5 Pa to determine their apparent viscosity (Pas) and shear stress (Pa). A frequency sweep was performed on gel samples with constant deformation (2% strain) in a frequency range of 0.1 to 20 Hz at 25 °C.

### 2.13. In Vitro Digestibility

The in vitro digestibility of all samples was tested using the procedure presented by Englyst et al. [17] with slight modification. Samples (50 mg) were accurately weighed and then dissolved in 20 mL sodium acetate buffer (0.2 M, pH 6.0). After being equilibrated at 37 °C for 10 min, they were mixed with a 5 mL enzyme suspension which was prepared with Porcine pancreatic alpha-amylase (20 mg) and amyloglucosidase (172 uL) in 5 mL of sodium acetate buffer. Lately, the mixture was continuously shaken in a water bath at 37 °C with 200 rpm. The aliquot (500 µL) of hydrolyzed fluid was removed at intervals (0, 10, 20, 30, 60, 90, 120, and 180 min), then quickly put into boiling water to terminate digestion. Subsequently, the mixture was then centrifuged at 4000 rpm for 10 min, and a Biosensors analyzer (S-10, Sieman Technology Co., Ltd., Shenzhen, China) was used to measure the amount of glucose in the supernatant. The following formulas were used to determine the amounts of rapidly-digestible starch (RDS), slowly-digestible starch (SDS), and resistant starch (RS):

$$\mathrm{RDS}\;\left(\%\right)=\left[\frac{G20-\mathrm{FG}}{T}\right]\times 0.9\times 100$$

$$\mathrm{SDS}\;\left(\%\right)=\left[\frac{G120-G20}{T}\right]\times 0.9\times 100$$

$$\mathrm{RS}\;\left(\%\right)=\left[\frac{T-\left(\mathrm{RDS}+\mathrm{SDS}\right)}{T}\right]\times 100$$

G20 and G120 stand for the contents of hydrolysis glucose within 20 and 120 min; FG is free glucose content; T refers to the content of total starch; 0.9 is the conversion factor.

### 2.14. Statistical Analysis

All statistical analyses were conducted using SPSS software (SPSS Inc., Chicago, IN, USA), and variance (ANOVA) was used to measure statistical differences. *p* < 0.05 represents statistically significant differences.

## 3. Results and Discussion

### 3.1. Solubility and Swelling Power (SP)

**Figure 1:**
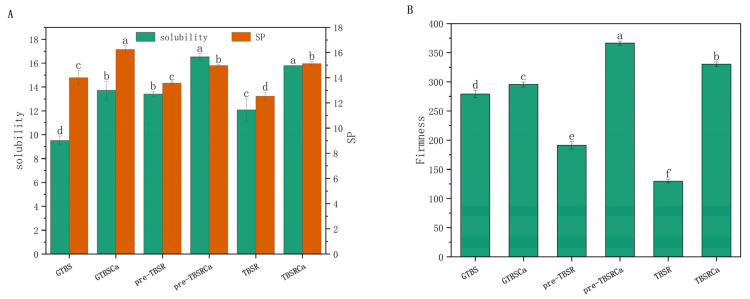
SP and solubility of each sample (**A**); gel firmness of heat-treated Tartary buckwheat starch and starch complexes for 1 day (**B**). Different lowercase letters within the same color column represent significantly different (*p* < 0.05).

Figure 1A shows the solubility and SP of all samples. It was observed that the presence of Ca(OH)_2_ and rutin greatly improved the solubility of each sample, while it was not significant between pre-gelatinization and co-gelatinization methods. TBSRCa has a higher solubility than GTBSCa and TBSR, either in pre-gelatinized or co-gelatinized samples. Compared to GTBS, the SP of TBSR decreased significantly, and the reduction of SP of pre-TBSR is not significant, while the SP of GTBSCa, TBSRCa, and pre-TBSRCa increased. It can also be seen that the SP of TBSRCa was higher than TBSR but lower than GTBSCa.

Starch-water interactions could explain the increased solubility. On the one hand, interactions between the hydroxyl groups of phenolic acids in water change the water activity and ionic strength of an aqueous solution. This alteration encourages the dissolution of soluble starch [18], and the particles of rutin are smaller and irregular, allowing for a larger specific surface area in the solution and thus having higher solubility [2]. On the other hand, the presence of Ca(OH)_2_ created pores on the starch surface and led more water and alkali into the starch, thus accelerating the protons’ separation from their hydroxyl groups on starch [11]; it might be that Ca^2+^ or Ca(OH)^+^ forms a complex with starch to destroy starch granules, leading to higher solution dispersion and solubility.

The decrease in SP might be attributed to the addition of rutin, which prevents the interaction between starch granules and amylopectin or amylose, limiting starch particle expansion force [2]. Zhang et al. [6] found similar results when studying the interaction of polyphenols with corn starch. On the other hand, the SP increased with the addition of Ca(OH)_2_; the formation of alkaline conditions in the presence of appropriate amounts of Ca(OH)_2_ promotes the release of amylose from starch granules and the release of hydroxyl groups from starch chains, which may result in Ca^2+^ and Ca(OH)^+^ proton substitution. Interactions between Ca^2+^, Ca (OH)^+^, and starch increases the swelling capacity of particles [11].

### 3.2. Gel Firmness

Textural property is an important indicator of the gel system, among which gel hardness is commonly used to assess the amount of starch retrogradation during storage. Figure 1B shows the firmness properties of all samples. As can be observed, the firmness of TBSR-1d and pre-TBSR-1d were significantly reduced compared to GTBS-1d, while the firmness of GTBSCa-1d, TBSRCa-1d and pre-TBSRCa-1d were significantly increased. Furthermore, there were significant differences between the two heating methods on the hardness of samples, and the pre-gelatinized complex had higher firmness than the co-gelatinized complex.

Compared with GTBS, the decrease in hardness of pre-TBSR and TBSR samples may be due to the fact that the hydroxyl group on the polyphenol molecular chain can combine with the amylose molecule, which inhibits the formation of amylose double helix and regeneration crystallization [19], thus leading to a weaker starch gel structure, which corresponds to the results of He et al. [7], the decrease in hardness suggested that the retrogradation of the starch gel had been reduced because firmness and starch retrogradation are closely related. The higher firmness value in samples containing Ca(OH)_2_ might be due to the role of Ca^2+^ ions, which may interact with starch chains that contribute to forming an interconnection network [20]. The gel firmness of pre-TBSRCa-1d was higher than that of other samples, which may be due to the presence of Ca(OH)_2_ as a cross-linker that enhanced the interaction between rutin and starch. At the same time, the firm gel structure can also be seen in SEM.

### 3.3. Morphological Characteristics

**Figure 2:**
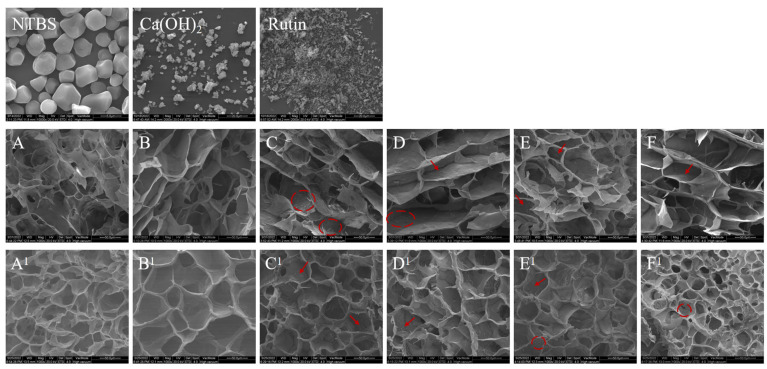
Scanning electron microscopy of nature TBS (NTBS), Ca(OH)_2_, rutin, gelatinized TBS (GTBS), GTBSCa, pre-TBSR, pre-TBSRCa, TBSR, and TBSRCa complexes gelatinization (**A**–**F**) and after regeneration (**A^1^**–**F^1^**).

The morphology characteristics of all samples, including Ca(OH)_2_ and rutin, are shown in Figure 2. Native Tartary buckwheat starch (NTBS) showed polygonal, near-spherical, or irregular shape particles, while rutin and Ca(OH)_2_ exhibited irregular solid particle aggregates. NTBS lost their native shape after hydrothermal treatment, and no residual starch granules can be observed in the gelatinized and retrograded samples, indicating that the present hydrothermal condition induced complete gelatinization of NTBS. All gelatinized (Figure 2A–F) and retrograded samples (Figure 2A^1^–F^1^) exhibited a three-dimensional honeycomb network structure with a multidimensional void wrapped in the gel wall. However, it could be observed that the homogeneity and continuity of the gel wall were visible differences in the different samples. In contrast to GTBS (Figure 2A), the complexes added with rutin (Figure 2C,F) could be discovered that the surface of the gel wall was rough and uneven, and rutin unevenly distributed in the cavity and pore wall of the gel, it may limit the interconnections among starch molecules, which resulted in some unconnected gel walls. This phenomenon is more obvious in co-gelatinized samples (Figure 2E,F). However, GTBSCa (Figure 2B) has a more uniform structure than GTBS (Figure 2A). The presence of Ca(OH)_2_ improved the situation; it reduced the unevenness and increased the connectivity of the gel walls. Retrograded samples presented similar trends to the gelatinized samples. Simultaneously, it also could be observed that retrograded complexes containing Ca(OH)_2_ (Figure 2B^1^,D^1^,F^1^) possessed a thicker gel wall than those samples without Ca(OH)_2_.

The morphology of NTBS and rutin observed during this investigation is in accordance with earlier literature [2,10]. Aleixandre et al. [21] indicated that differences in the wall thickness depend on the starch source and their chemical composition. Compared to GTBS, more unconnected gel walls of the complexes with the addition of rutin might be attributed to the rutin particles, which were unevenly distributed in the gel wall and prevented starch aggregation [2]. Consequently, in the process of TBS particles absorbing water and expanding, the precipitation particles dispersed in the starch matrix prevent the contact of starch molecules, destroy the orderly arrangement of starch molecules, and limit the formation of gel [2]. The present results indicated that the complex of starch and rutin possessed a more loose and porous structure, which was also demonstrated by He [7], Zhu [22] and Wang et al. [23]. The thickening of the gel wall may be due to the deposition of rutin in the gel wall or the interaction between starch and rutin. The addition of Ca(OH)_2_ in TBS enhanced the formation of the hydrogel microarchitecture of GTBS, which was consistent with the findings of Cornejo–Villegas [13], who indicated that adding Ca(OH)_2_ to gelatinized starch formed a filamentous network and promoted the formation of the hydrogel microstructure. The presence of Ca(OH)_2_ in complexes of TBS and rutin smoothed and firmed the gel walls might be because that alkaline condition promotes the dissolution of rutin and makes it easily permeate into the starch matrix and interact with starch, generating a smoother and more compact starch reticular structure. During the preparation of the pre-gelatinized complex, rutin might be easier to deposit on the gel wall, rather than prevent the interaction of starch molecules, resulting in the pre-gelatinized complex of TBS and rutin showed the thicker and continue gel wall than co-gelatinized complex.

In short, the present finding from SEM indicated rutin was dispersed in a starchy matrix and hindered the formation of continuous gel walls. Ca(OH)_2_ facilitated the dispersion, smoothed the surface, and firmed the gel walls.

### 3.4. Thermogravimetry Analysis

**Figure 3:**
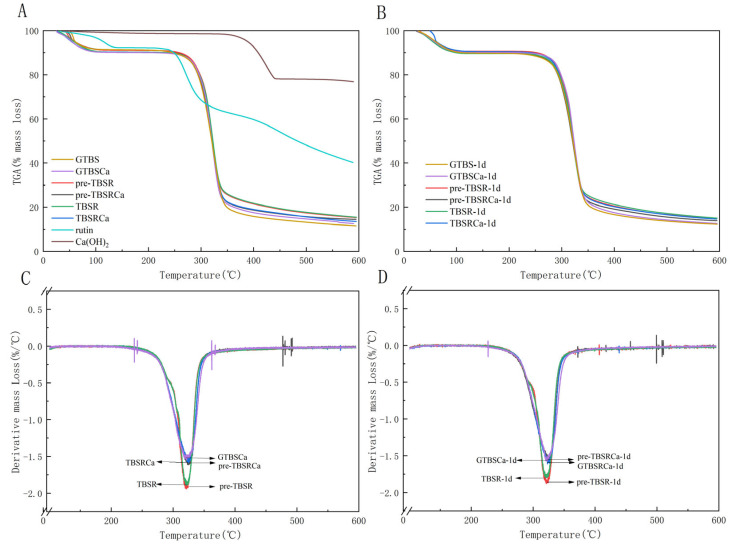
TGA curves of gelatinized (**A**) and retrograded (**B**) samples; DTG curves of gelatinized (**C**) and retrograded (**D**) samples.

Figure 3A,B shows the TGA patterns of all samples, including the gelatinized samples and retrograded samples for 1 day at 4 °C. 

According to Figure 3A,B, the thermal decomposition process of Ca(OH)_2_ showed only one stage of decomposition around 400 °C, which is different from that of complexes. The thermal decomposition processes of complex prepared with pre-gelatinized and co-gelatinized exhibited two main steps. The first decomposition processes occurred immediately after warming up and ended before 200 °C. The second mass loss happened between 250–400 °C with the mass of GTBS decreasing from 90% to around 20%, and from 90% to around 26% for TBSR and pre-TBSR, while the mass of TBSRCa, pre-TBSRCa, and GTBSCa was reduced from 90% to around 23%. The remaining rate of the rutin and Ca(OH)_2_ was above 40% and 80% when the temperature reached 600 °C, while decreased to around 20% after adding into starch, indicating the formation of starch-rutin, starch-Ca(OH)_2_ and starch-rutin-Ca(OH)_2_ complex. DTG curves in Figure 3C,D shows that the temperature of maximum weight loss (T_max_) of GTBSCa, TBSR, and TBSRCa was 320 °C, 321 °C, and 325 °C, respectively, either in pre-gelatinized or co-gelatinized samples, indicating an increase in their thermal stability in the presence of Ca(OH)_2_ and rutin. Retrograded samples presented similar trends to the gelatinized samples.

The first stage of mass loss represents the removal of water and low molecular compounds present in the samples. Starch molecules contain some water, and the hydrophilicity might be due to the hydrogen bond formed by the hydroxyl group of the glucose unit [24]. The second stage of mass loss indicates that starch decomposition corresponds to the elimination of hydroxyl groups, as well as carbon chain breakdown and depolymerization [25]. Correspondingly, the increase of T_max_ indicated an increase in the sample’s thermal stability in the presence of Ca(OH)_2_ and rutin, which were also reported in a previous study [15], which found that the mass loss of starch complex was reduced and its thermal stability was improved after quercetin treatment. Moreover, the T_max_ of the TBSRCa is slightly higher than that of the TBSR and GTBSCa, indicating that the TBSRCa loses less weight and has better thermal stability at the same temperature [6].

### 3.5. Crystalline Structure and Crystallinity

**Figure 4:**
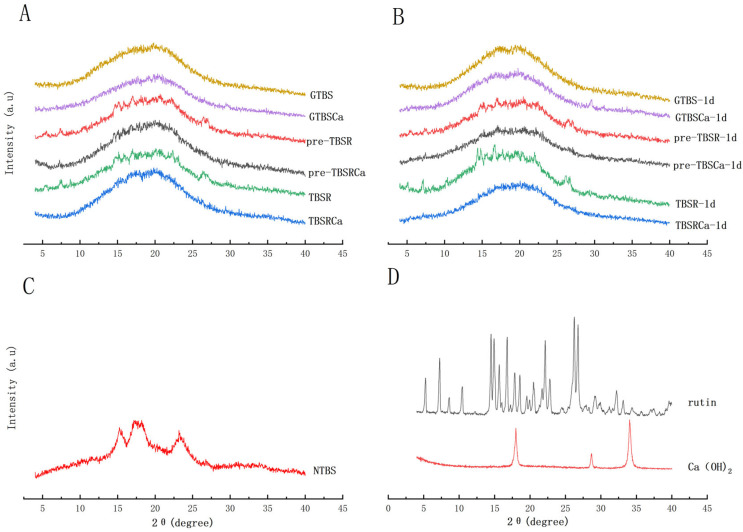
X-ray pattern of GTBS, GTBSCa, pre-TBSR, pre-TBSRCa, TBSR and TBSRCa complexes (**A**) and retrograded samples for 1 day (**B**); NTBS (**C**); Ca(OH)_2_; rutin (**D**).

Figure 4 shows the X-ray diffraction patterns of each sample, and relative crystallinity (RC) is displayed in Table 1. It was shown that rutin displayed a certain crystalline structure. Strong diffraction peaks at 15.0°, 17.1°, 18.0°, and 24.0° were present in the NTBS, which displayed typical A-type crystallinity, whereas GTBS exhibited an amorphous pattern with broad peaks, and the RC of GTBS (7.41%) was significantly lower than NTBS (32.08%).

An amorphous pattern with broad peaks was observed either in pre-gelatinized or co-gelatinized samples. The rutin crystalline peak can be seen at 26.43° in pre-TBSR and TBSR, but its intensity decreased in pre-TBSRCa and TBSRCa. Table 1 also showed that the RC of other complex gels was significantly lower than that of GTBS at the same retrogradation time. The RC of GTBS increased rapidly from 7.41% to 10.04% (storage for 1 day), while the RC of TBSR and TBSRCa increased slowly, only from 5.41% to 6.81% (storage for 1 day) for TBSR and from 5.35% to 6.64% (storage for 1 day) for TBSRCa, which showed the slowest increase during storage.

As evaluated by X-ray diffractometry, the original crystalline shape of the gelatinized and regenerated starch was lost, but the presence of Ca(OH)_2_ and rutin had no effect on the crystalline shape of the pasted starch, which is consistent with previous reports on buckwheat starch [2,13]. After processing, the typical diffraction peak of rutin at 26.43° was significantly weakened, indicating that rutin migrated into starch [15]. Roskhrua et al. [26] also suggested that the sample treated with alkali merely modifies the diffraction peak’s intensity rather than the amylopectin in starch granules’ crystallization pattern, leading to a decrease in RC of samples [27], similar to the findings of our study. The addition of Ca(OH)_2_ and rutin affects the starch retrogradation to various degrees, whether pre-gelatinization or co-gelatinization. At the same storage time, both TBSRCa and TBSRCa -1d had the lowest RC values, and the RC of gelatinized complexes grew slowly compared to gelatinized starch, implying that the presence of Ca(OH)_2_ and rutin may inhibit the formation of recrystallization of starch, delays the retrogradation of GTBS.

### 3.6. FT-IR Analysis

**Figure 5:**
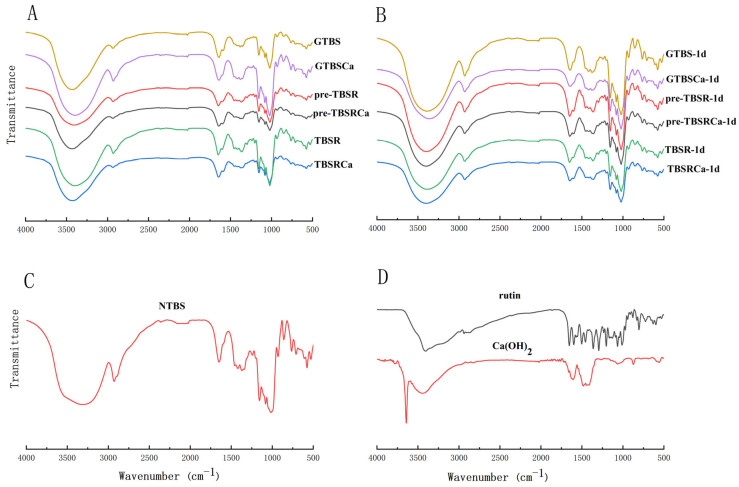
FTIR spectra of the GTBS, GTBSCa, pre-TBSR, pre-TBSRCa, TBSR and TBSRCa complexes (**A**) and retrograded samples for 1 day (**B**); NTBS (**C**); rutin and Ca(OH)_2_ (**D**).

Infrared spectroscopy is sensitive to chemical bond oscillation frequency, and vibrational modes are related to starch conformation and crystallographic order [28]. The FT-IR spectra were used to investigate the proximal order and helical structures of all samples under two processing methods, as well as the FT-IR spectral variations in the 400–4000 cm^−1^ band of all samples were determined, as shown in Figure 5. The ratio of 1047/1022 cm^−1^ (the degree of order, DO) [29] was displayed in Table 1.

According to Figure 5A,B, the complexes had similar absorption peaks. The broad absorption peak at 3300 cm^−1^ indicates stretching of the -OH group in starch or phenolics, which could be observed in all complexed and GTBS. The OH^−^ stretching in Ca(OH)_2_ is represented by the absorption peak at 3642 cm^−1^, which only was found in the Ca(OH)_2_, and could not be observed in the complex added with Ca(OH)_2_. The absorption peak at 1650 cm^−1^ is associated with C-O-O stretching vibration of carbohydrates, and the absorption peak at 2930 cm^−1^ is associated with the C-H stretching vibration [2]. At the same time, no discernible change was found in the vibration among starch and starch complexes. The spectral bands at 1022 cm^−1^ were related to the vibrational modes of the amorphous regions of starch, whereas the bands at 1022 and 1047 cm^−1^ were correlated with the number of ordered regions [30]. The DO values of TBSR, GTBSCa, and TBSRCa were lower than GTBS, while the pre-gelatinized complex had higher DO values than the co-gelatinized complex, even if DO values increased in the samples of retrogradation for 1d. Present results indicated that Ca(OH)_2_ and rutin interfere with the formation of ordered structures in the starch crystal region. Chai et al. [31] also observed similar results when they investigated the complex of starch with tea polyphenols.

It was noticed that there were no new characteristic peaks in TBSR or pre-TBSR, which demonstrated that the rutin would not lead to the formation of new chemical bonds in starch samples [2]. Furthermore, previous research revealed that the addition of Ca^2+^ ions does not form new chemical bonds in the starch matrix, but the Van der Waals interactions might affect the physicochemical characteristics of starch [32,33].

### 3.7. Differential Scanning Calorimetry

The parameters of DSC with all samples were observed in Table 2. Compared to GTBS, the peak temperature (T_P_) values of GTBSCa were significantly higher by about 4°C, while the gelatinization enthalpy (ΔH) value decreased from 9.44 to 7.09. Compared to GTBS, the onset temperature (T_O_), T_P_, conclusion temperature (T_C_), and ΔH values of TBSR and pre-TBSR were decreased, the T_O_, T_P_, and T_C_ values of TBSRCa and pre-TBSRCa were higher than GTBS, TBSR, and pre-TBSR and were lower than GTBSCa, and the ΔH value of TBSRCa was the lowest among the starch complexes. We found that the change in trends of retrogradation enthalpy (ΔHr) was consistent with the ΔH, the ΔHr value of all complexes was significantly lower than that of GTBS-1d, and the ΔHr value of TBSRCa-1d was the lowest. Meanwhile, the value of the co-gelatinized sample was lower than that of the pre-gelatinized sample.

Wang et al. [2] found that the T_O_, T_P_, and T_C_ values of the starch sample decreased with the addition of rutin, and ΔH was reduced to different degrees, implying that the starch gel was less susceptible to retrogradation under the presence of rutin. Previous research found that alkali treatment can increase the gelatinization temperature of starch and reduce ΔH value [32], which is similar to our research results. The increase in T_O_ indicates that the melting of starch granules starts at a higher temperature [34]. Ca(OH)_2_ dissociates into Ca^2+^ and OH^−^ ions in the process; the higher the amount of OH^−^ anions, the easier it is to penetrate the starch granule and destroy the hydrogen bond in the starch chains, which enhances the gelatinization process [35]. ΔH represents the energy required to disorder amylopectin in granules for gelatinization [36]. The addition of Ca(OH)_2_ and rutin reduced the enthalpy of gelation of TBS, implying that they reduced the energy required to change from suspension to gelation state. Moreover, TBSRCa had the lowest ΔH, which may be caused by the connection among starch, rutin and Ca(OH)_2_. The lower ΔH of complexes indicated that the presence of Ca(OH)_2_ and rutin inhibited TBS retrogradation. The starch complexes showed obviously lower ΔHr, which was similar to the trend of starch enthalpy reported by Tang et al. [37], that the decrease of enthalpy may be due to the restriction of the combination of amylose and amylopectin. The ΔHr of all samples is lower than GTBS, indicating that the presence of rutin and Ca(OH)_2_ is beneficial to delay the buckwheat starch’s retrogradation, as well as co-gelatinization has a better effect.

### 3.8. Rheological Properties

**Figure 6:**
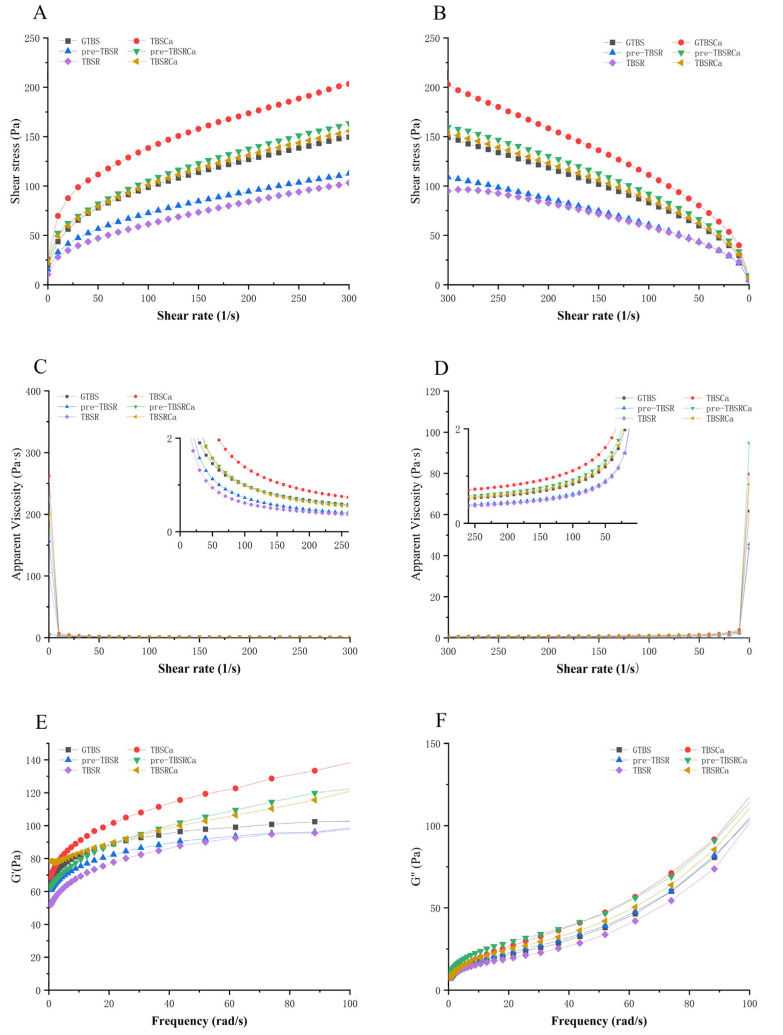
Upward (**A**,**C**) and downward (**B**,**D**) flow curves of samples; storage modulus G′ (**E**); loss modulus G″ (**F**).

The rheological properties of all the samples are depicted in Figure 6. As shown in Figure 6A–D, The apparent viscosity decreases with the increase of shear rate during the process of upward shearing (Figure 6C), which indicates that the flow curves of each sample displayed pseudo-plasticity and shear thinning features, regardless of pre-gelatinization or co-gelatinization. Compared with GTBS, TBSR and pre-TBSR have lower apparent viscosities, while there is no significant difference among samples prepared with the pre-gelatinization and co-gelatinization methods. With the addition of Ca(OH)_2_, the apparent viscosities of TBSRCa, pre-TBSRCa, and GTBSCa were higher than that of GTBS, while GTBSCa has the highest apparent viscosity, which could be attributed to the formation of a strong and continuous network, resulting in an improvement in the consistency of the starch complexes [15].

Figure 6E,F showed the dynamic rheology of all samples. The values of storage modulus (G′) and loss modulus (G″) are frequently used to characterize the rigidity and toughness of the recently formed starch gel network [38]. Both G′ and G″ values were gradually increased with the increasing angular frequency. Compared with GTBS, the decrease of G′ and G″ of TBSR and pre-TBSR indicates that rutin will dilute the starch paste, leading to the destruction of the gel structure; a similar phenomenon has also been found in the research of Wang et al. [2], who revealed that precipitates of rutin at a concentration of greater than 7% diluted the starch pastes and caused structural disruption of the gel. However, TBSRCa, pre-TBSRCa, and GTBSCa have higher G′ and G″ values than GTBS, which proves that the addition of the complex forms a strong cross-linked gel network. Similar to our results, Santiago–Ramos et al. [35] have found that calcium hydroxide treatment will increase the G′ and G″ values of masa from corn flours, which may form a calcium starch cross-linking structure, resulting in a larger gel network. The loss angle (tan δ) is equal to G″/G′. The higher tan δ of the starch-rutin complex indicates that the sample shows higher fluidity [36], which may be caused by the interaction between rutin and starch inhibits the formation of a gel network or restricts the leaching of amylose, thus forming a weaker gel [39]. However, the starch-Ca(OH)_2_ complex has a low tan δ value, indicating that Ca(OH)_2_ will affect the rheological behavior of the starch-rutin complex, and Ca(OH)_2_ may be beneficial to the release of amylopectin, thus affecting its viscoelasticity [35]. The assumption goes that an appropriate amount of rutin and Ca(OH)_2_ are used as molecular chaperones to assist in the reorganization of starch, which may form a starch network or a starch-rutin-Ca(OH)_2_ architecture.

### 3.9. In Vitro Digestibility

**Figure 7:**
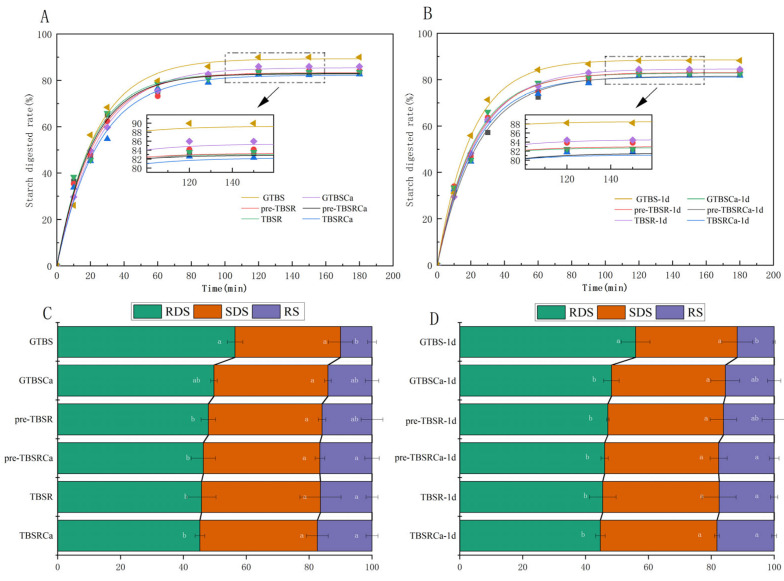
Starch hydrolysis pattern of GTBS, GTBSCa, pre-TBSR, pre-TBSRCa, TBSR, and TBSRCa complexes (**A**) and retrograded samples for 1 day (**B**); the starch fraction of samples (**C**) and retrograded samples for 1 day (**D**). Different lowercase letters in columns of the same color indicates significant differences (*p* < 0.05) of same starch fractions among samples.

Interactions between starch and other food ingredients have been shown to influence in vivo starch digestion and postprandial blood glucose response. In vitro digestibility changes of all samples were diagrammed in Figure 7. The digestive qualities of starch can be reflected in changes to RDS, SDS, and RS values [15,40].

From Figure 7A,B, it was observed that the digestibility of GTBS is higher than those of complexes addition with Ca(OH)_2_ and rutin, indicating that the in vitro digestibility of GTBS could be greatly impacted by rutin and Ca(OH)_2_. Similar digested results were displayed in retrograded samples. The change of digestibility is as follows: the starch sample without any substance has the highest digestion rate, the digestion rate of samples treated with rutin or Ca(OH)_2_ was significantly lower than GTBS, and TBSRCa was the lowest. Moreover, the digested rate of complexes with the pre-gelatinization method is slightly higher than that of the co-gelatinization method.

The contents of the RDS, SDS and RS complexes are presented in Figure 7C,D. The RDS, SDS, and RS contents of GTBS were 56.42%, 33.56%, and 10.02%, respectively. In contrast to GTBS, pre-gelatinized and co-gelatinized complexes showed lower contents of RDS and higher contents of SDS and RS. The RDS contents of TBSRCa and pre-TBSRCa were 45.25% and 46.34%, respectively. The RS content of TBSRCa was 17.40%, which was the highest among the complexes. TBSRCa and pre-TBSRCa had lower RDS and SDS values than GTBS, indicating that the addition of Ca(OH)_2_ and rutin promoted the conversion of RDS and SDS to RS in TBS. The same changes were applied to the retrograded samples. Wang et al. [41] studied the effect of rutin on the digestion of TBS and found that the addition of rutin can significantly reduce the content of SDS of complex, increase the content of RS, and the digestibility of complex is significantly lower than GTBS. Rutin can reduce starch digestion by changing the structure of the combined form of starch and inhibiting the free form of digestive enzyme activity [41]. Gao et al. [15] also found that quercetin has a limited inhibitory effect on amylase under hydrothermal conditions, and the formation of the complex may also be a reason for inhibiting its digestibility. Zeng et al. [42] found that the addition of tannic acid significantly reduced the digestibility of starch, and the content of RDS and SDS decreased, while the content of RS increased, which is similar to our research results. Present results indicated that the formation of TBS-rutin-Ca(OH)_2_ complexes is most likely the primary cause of the reduction in starch digestibility.

## 4. Conclusions

The effect of Ca(OH)_2_ on structural, physicochemical, and in vitro digestibility properties of complex of TBS and rutin were investigated in this study, and pre -gelatinization and co-gelatinization methods were compared. The present study has shown that Ca(OH)_2_ promoted the connection of the three-dimensional network structure of gelatinized and retrograded TBS-rutin complex and effectively inhibited the retrogradation of TBS. TBSRCa possessed lower RC, DO and enthalpy values, lower digestibility, and higher content of RS compared to other samples. The rheological results also showed that TBSRCa modified the viscoelastic behavior of starch pastes. Compared with pre-gelatinization, complexes prepared with the co-gelatinization method presented better properties.

In short, the addition of Ca (OH)_2_ and rutin is conducive to the gelatinization of TBS, and Ca(OH)_2_ treatment should be taken into consideration as a potentially effective method for preparing starch-polyphenols complexes. In the future, more research using modern analytical means is needed to clarify the formation mechanism of starch-polyphenol complexes in the presence of calcium hydroxide at the molecular structure level and bond valence level.

## Figures and Tables

**Table 1:** Relative crystallinity (RC) and the ratio of 1047/1022 cm^−1^ (DO) of samples.

	RC (%)	RC (%)-1d	DO	DO-1d
GTBS	7.41 ± 0.05 a	10.04 ± 0.03 a	1.17 ± 0.006 a	1.27 ± 0.008 a
GTBSCa	7.10 ± 0.11 b	8.54 ± 0.1 b	1.17 ± 0.005 a	1.23 ± 0.006 b
pre-TBSR	5.98 ± 0.04 c	7.01 ± 0.03 c	1.10 ± 0.005 b	1.18 ± 0.004 c
pre-TBSRCa	5.60 ± 0.03 d	6.81 ± 0.03 d	1.05 ± 0.002 e	1.14 ± 0.004 d
TBSR	5.41 ± 0.05 e	6.81 ± 0.13 d	1.08 ± 0.001 c	1.13 ± 0.004 e
TBSRCa	5.35 ± 0.05 e	6.64 ± 0.07 e	1.07 ± 0.002 d	1.08 ± 0.002 f

Different lowercase letters in the same column are significantly different (*p* < 0.05).

**Table 2:** Pasting and gelatinization property changes of samples.

	T_O_ (°C)	T_P_ (°C)	T_C_ (°C)	△H (J/g)	△Hr (J/g)
GTBS	60.69 ± 0.07 d	67.17 ± 0.02 d	74.57 ± 0.3 b	9.44 ± 0.34 a	2.21 ± 0.09 a
GTBSCa	65.56 ± 0.05 a	71.11 ± 0.1 a	77.34 ± 0.09 a	7.09 ± 0.12 b	1.92 ± 0.07 b
pre-TBSR	60.43 ± 0.08 e	66.12 ± 0.09 e	72.40 ± 0.25 c	7.03 ± 0.08 bc	1.57 ± 0.03 c
pre-TBSRCa	63.78 ± 0.04 b	68.50 ± 0.17 b	74.61 ± 0.13 b	5.89 ± 0.09 d	1.27 ± 0.04 e
TBSR	60.21 ± 0.05f	66.11 ± 0.26e	72.16 ± 0.27c	6.61 ± 0.23c	1.41 ± 0.04d
TBSRCa	63.56 ± 0.06 c	67.98 ± 0.04 c	74.60 ± 0.14 b	5.24 ± 0.31 e	1.12 ± 0.01 f

T_O_, onset temperature; T_P_, peak temperature; T_C_, conclusion temperature; ΔH, gelatinization enthalpy; ΔHr, regeneration enthalpy. Data display average ± standard deviation. Different lowercase letters in the same column are significantly different (*p* < 0.05).

## Data Availability

Data is contained within the article.
